# Neurocognitive functions and social functioning in young females with recent-onset anorexia nervosa and recovered individuals

**DOI:** 10.1186/s40337-017-0137-3

**Published:** 2017-02-27

**Authors:** Mette Bentz, Jens Richardt Moellegaard Jepsen, Gry Kjaersdam Telléus, Ulla Moslet, Tine Pedersen, Cynthia M. Bulik, Kerstin Jessica Plessen

**Affiliations:** 1 Child and Adolescent Mental Health Centre, Mental Health Services in the Capital Region of Denmark, Bispebjerg Bakke 30, 2400 København NV, Denmark; 2 Department of Clinical Medicine, Faculty of Health and Medical Sciences, University of Copenhagen, Copenhagen, Denmark; 3 Lundbeck Foundation Center for Clinical Intervention and Neuropsychiatric Schizophrenia Research (CINS) and Center for Neuropsychiatric Schizophrenia Research (CNSR), Psychiatric Center Glostrup, Glostrup, Denmark; 4 Unit for Psychiatric Research, Aalborg University Hospital, Aalborg, Denmark; 5 Department of Clinical Medicine, Faculty of Medicine, Aalborg University, Aalborg, Denmark; 6 Danish Research Centre for Magnetic Resonance, Centre for Functional and Diagnostic Imaging and Research, Copenhagen University Hospital Hvidovre, Hvidovre, Denmark; 7 Department of Psychiatry, University of North Carolina at Chapel Hill, Chapel Hill, USA; 8 Department of Nutrition, University of North Carolina at Chapel Hill, Chapel Hill, USA; 9 Department of Medical Epidemiology and Biostatistics, Karolinska Institutet, Stockholm, Sweden

**Keywords:** Anorexia nervosa, Recovered, Adolescents, Neurocognition, Verbal memory, Social function

## Abstract

**Background:**

Young individuals with anorexia nervosa (AN) or recovered from AN display impairments of social function. To date, however, it is not clear whether they differ from controls with respect to neurocognitive performance and whether those functions contribute to the compromised social function observed in individuals with AN.

**Methods:**

We included 43 young females with first-episode AN, 28 individuals recovered from adolescent-onset AN, and 41 control individuals (14–22 yr), all without comorbid autism spectrum disorder. We compared the performance of participants across groups in seven neurocognitive functions relevant to social functioning: set-shifting, local processing, processing speed, working memory, sustained attention, verbal memory, and verbal abstraction. Further, we tested the association between neurocognitive function and social function, measured by Autism Diagnostic Observation Schedule (ADOS), with an ordinal logistic regression model.

**Results:**

First, participants did not differ on any neurocognitive function across groups. Second, only the neurocognitive function “verbal memory” was significantly associated with social function. Higher performance in verbal memory was associated with lower odds of impaired social function. Diagnostic group remained a significant factor, but the absence of an interaction between group and neurocognitive performance indicated that the association between verbal memory and social function was independent of group membership.

**Conclusion:**

Young individuals with AN and those recovered from AN did not differ from controls with respect to neurocognitive performance. Verbal memory was associated with social function in all groups.

## Plain English Summary

Difficulties in interacting with others are more common among young persons with anorexia nervosa than among their peers, and the same holds for youth who have recovered from anorexia nervosa. We thus investigated whether young individuals with anorexia nervosa and those recovered from anorexia nervosa showed difficulties in specific areas of mental abilities: the ability to switch between tasks and strategies, the tendency to focus overly on details, speed of problem solving, the ability to keep information in mind when needed, memory for spoken information, the ability to stay attentive, and finally the ability to interpret sayings in a non-literal fashion. We further investigated whether performance in these domains was associated with the capacity for social interaction in these groups. We found a normal level of functioning in all mental tasks. Moreover, participants with less accurate memory for spoken information were more likely to show difficulties in social interaction. This information might be useful in planning treatments that are helpful for the individual patient in the future.

## Background

Anorexia nervosa (AN) is a life-threatening psychiatric disorder characterized by distorted body image and a persistent restriction of food intake leading to low body weight, often by applying inflexible rules and rigid behaviors around eating [1, 2]. Despite a growing body of research, the etiology of AN is not mapped out clearly, and success rates of available treatments are modest. A contemporary model of AN, the social-emotional maintenance model, [3, 4] proposes that an interaction between intrapersonal and interpersonal factors, reinforced by the effects of starvation, maintains the disorder. Moreover, this model highlights the importance of neurocognitive deficits, especially inflexibility and exaggerated attention to detail, and deficits in social cognition, for the emergence and the maintenance of anorectic symptoms [4].

We have previously observed impaired social functioning in young females with first-episode AN without autism spectrum disorder (ASD), [5] when assessing social functioning with a semi-structured, standardized assessment of social interaction, which emphasizes communication and socio-emotional reciprocity [6]. Moreover, young females fully recovered from childhood or adolescent-onset AN [5] displayed a similar degree of impairment in social function suggesting that these inefficiencies do not improve with recovery. To date, it is not clear, however, which factors determine the presence and the degree of difficulties of social function in young individuals with AN.

Evidence from other disorders suggests that specific neurocognitive functions play a role in the capacity of social function. For instance, a broad range of neurocognitive functions predicts approximately a quarter of the variance in social functioning among individuals with schizophrenia [7]. Further, impairments of social and executive functions co-occur and interact in individuals with ASD [8, 9]. Finally, a prospective study of adolescents with AN reported that individuals with co-existing neurocognitive deficits and ASD-traits had a lower rate of recovery from AN [10–12].

Adults with AN display impairments of social functioning (e.g. as defined by the RDoC [13] domain “Systems for Social Processes”) [14, 15]. Moreover, cognitive inflexibility and a cognitive style of superior local processing in the context of weak central coherence have consistently been reported in adults with AN [16, 17]. These findings inspired the hypothesis that cognitive inflexibility and weak central coherence may represent possible endophenotypes of AN [18–21]. The fact, however, that adolescents with AN display normal levels of flexibility, as well as central coherence [22, 23] calls for further inquiring the trait-like nature and the continuity of these findings.

Considerable neurocognitive heterogeneity may exist within adolescents and adults with AN, both within and across the age groups. A cluster analysis in children and adolescents with AN reported three distinct clusters of neurocognitive profiles [24]. A cluster analysis in adults with AN also documented distinct subgroups, one of which displayed co-occurrence of neurocognitive and social-cognitive impairment [25]. Other lines of evidence suggest that adolescents with AN may display subtle cognitive inefficiencies. In a large cohort study, adolescents with AN performed lower in a nonverbal aspect of intelligence (Perceptual Organization Index), [26] and in verbal memory, [27] even after weight recovery [28]. These participants, however, displayed normal general intelligence and normal set shifting ability. Subtle differences in neurocognitive functioning may thus exist in adolescents with AN, [29–31] or even in subgroups, despite their function being similar to controls on most neurocognitive measures [22, 23, 31, 32].

Hence, the impairments of social functioning previously observed in young individuals with AN [5] may relate to subtle inefficiencies in cognitive flexibility, in central coherence ability or in other neurocognitive functions. Moreover, within-group associations may exist between social functioning and those neurocognitive functions in subgroups of the population, despite the observation that individuals with AN on the group level display normal neurocognitive performance. Correlations of social functioning and specific neurocognitive deviations thus may contribute to understand the impairment of social functioning in more detail.

Potential associations between neurocognitive functions and social functioning, however, have not been investigated to date in adolescents with AN. This gap of evidence concerning adolescents with AN is surprising, because knowledge of such associations could potentially lead to interventions that target enhancing social function, e.g. by remediating therapy in the subgroup of young individuals with AN with neurocognitive inefficiencies. Social functioning in adolescence may be more vulnerable to the effect of inefficient neurocognitive functions, because interactions with peers become increasingly complex, indirect and fluctuating, and follow less explicit social rules compared to the more unequivocal rules of play in childhood [33].

Thus, the overall purpose of the present study was to further understand the observed impairment of social functioning by investigating potential associations with neurocognitive functions in these young diagnostic groups. We aimed to: (i) compare neurocognitive functioning in young individuals with first-episode and in those recovered from AN with controls, and (ii) test whether specific neurocognitive functions contributed to the variation of social functioning previously reported in the same population [5].

We hypothesized first, that young individuals with first-episode AN and recovered individuals would display deficits in aspects of neurocognitive function, and second, that several neurocognitive functions in the young individuals with first-episode AN and those recovered would be associated with social function.

## Methods

### Participants

We included young persons between 14 and 22 years in the study. Participants in the first-episode AN group were young females with a recent onset of their first episode of AN (International Classification of Diseases (ICD-10): F50.0 or F50.1),[1] consecutively invited to participate when presenting for treatment in the Mental Health Services, Capital Region of Denmark. Individuals in the recovered group had an onset of AN in late childhood or adolescence (ICD-10: F50.0 or F50.1) [1] and were invited during participation in a clinical follow-up. Control participants were recruited via notice board advertisements in schools, halls of residence, and colleges in the catchment area of the hospital. Inclusion criteria for participants with first-episode AN were underweight, defined as BMI ≤ 18.5 kg/m^2^ for participants older than 16 yr and a BMI-percentile corrected for age ≤ 25th percentile in those 14–15 yrs [34]. Inclusion criteria for participants recovered from AN were maintenance of normal body weight (defined as BMI > 18.5 kg/m^2^ for participants older than 16 yr and a BMI-percentile corrected for age > 25th percentile in those 14–15 yrs) for a minimum of one year, no present eating disorder pathology, a global score of the Eating Disorders Examination (EDE) within one standard deviation (SD) of non-AN mean, [35] and a generally favorable outcome with a score of ≥ 9 on the Morgan Russell Outcome Assessment Schedule (MROAS) [36]. Participants with infantile autism (F84.0) or Asperger’s syndrome (F84.5) were excluded, to ensure that our findings were representative of young individuals with AN and not explained by a few outliers with comorbid ASD. Participants with any past or present major psychiatric disorder were excluded in the control group (exceptions were mild episodes of transient tics earlier in childhood, or brief episodes of adjustment disorder after traumatic loss). Current treatment with psychotropic drugs was an exclusion criterion for individuals with first-episode AN and controls. We did not deem this criterion to compromise the representativeness of individuals with first-episode AN, because the use of psychotropic drugs is not typical in the early phases of treatment in young participants in our services. In contrast, we did not exclude recovered participants with current psychopharmacological treatment, because those individuals often experience other comorbid psychiatric disorders, such as anxiety and depression for which they receive medication [11, 37]. Comorbidity was assessed with the Schedule for Affective Disorders and Schizophrenia for School-Age Children, Present and Lifetime version (K-SADS-PL), [38] and the Beck Youth Inventory (BYI), [39] the latter yielding dimensional measures of anxiety (BAI-Y) and depression (BDI-Y). We assessed general intelligence with the Reynolds Intellectual Assessment Scales (RIAS), Danish version [40].

### Measures

#### Social functioning

We assessed social functioning with the Autism Diagnostic Observation Schedule (ADOS-2), Danish version, module 4, [6] in which a higher score reflects a higher degree of deviation of social and communicative behaviors. We used the combined scale “Communication and Social Interaction Total” (ADOS-Total) as the outcome measure of social functioning [5].

### Neurocognitive function

We assessed a range of neurocognitive functions that have consistently been associated with social functioning among individuals with AN or other disorders: [8, 12, 17, 22, 41] set-shifting, local processing bias, processing speed, working memory, sustained attention, verbal memory and verbal abstraction. For some neurocognitive functions, we chose a single test when this test is regarded as a relevant measure for a specific function, e.g. sustained attention. However, we combined tests into composite measures in the cases where several underlying aspects together captured complex structures, e.g. working memory and set-shifting / mental flexibility.

### Set-shifting (mental flexibility)

*The Delis-Kaplan Executive Function System* (D-KEFS) [42] is a battery of nine co-normed tests of executive functions. Each test consists of several subtests with separate scores. We employed the shift-conditions of Verbal Fluency (condition 3: generating words while alternating between two semantic categories), Design Fluency (condition 3: generating designs while alternating between black and white dots), and Trail Making (condition 4: alternating between digits and letters). We compared groups on the scores of these specific shift conditions rather than on the secondary contrast scores provided in D-KEFS, because the reliability of these has been questioned [43]. These three scores were combined into a set-shifting composite measure of cognitive flexibility. The combination of shifts between over-learned (Trail Making) and novel visual (Design Fluency) and novel semantic material (Word Fluency) was intended to cover the complexity of set shifting and potentially increase the sensitivity of the composite score [17].

### Local processing

*Group embedded Figures Test* (GEFT) [44] measures local processing with simpler shapes hidden in 18 complex geometric designs, and it benefits from a detail-focused processing style. We administered GEFT individually, timing each search,[45] and provided a separate sheet with target shapes to eliminate the reliance on memory [46]. The outcome measure was the number of shapes identified correctly within 120 seconds for each design (Table 2).

### Processing speed

We used 3 non-shift conditions of the Trail Making Test: condition 1 (Visual Scanning), 2 (Number Sequencing) and 3 (Letter Sequencing) as a composite measure of processing speed [42].

### Working memory

We assessed working memory with a composite score formed by three verbal and two nonverbal subtests: *Paced Auditory Serial Addition Test* (PASAT), Danish version, part 2, [47] Digit Span and Letter-Number Sequencing from *The Wechsler Adult Intelligence Scale* (WAIS-IV), Danish version [48], and *CANTAB® Spatial Working Memory* (SWM) and Spatial Span (SSP) [49]. We used total errors in the most difficult 8-box problems of SWM and span length of the SSP as outcomes. We included tests of working memory because it was associated with social-cognitive skills in a long-term follow-up study of adolescents with AN [50].

### Sustained attention

We used the total response time variability of the *Test of Variables of Attention* (TOVA) visual [51] as outcome measure assessing sustained attention. TOVA is a 22-minute computerized continuous-performance test.

### Verbal memory

We assessed verbal memory with a composite of the immediate and the delayed version of the subtest Memory for Stories from *Tests of Memory and Learning* (TOMAL-2), Danish version [52]. We administered story 3 and 4 of Memory for Stories to all participants regardless of age and compared raw scores across groups. The Tomal-2 Memory for Stories Test was sensitive to differences between adolescents with AN and controls in a large study [27].

### Verbal abstraction

The ability to form novel, verbal abstractions was measured with D-KEFS Proverbs condition 1, which requires the participant to explain in her own words the abstract meaning of a series of proverbs [42].

### Statistical procedures

We evaluated the effect of group on the seven neurocognitive functions in a within-participant repeated measure design, by applying a mixed effects model. We tested equal group effect on the seven different neurocognitive functions (parallelism) by evaluating the interaction between group and type of neurocognitive function, the latter as a categorical variable with seven levels. If the hypothesis of parallelism could not be rejected, we tested a hypothesis of similar mean scores across neurocognitive functions by evaluating the main effect by group. All tests were likelihood ratio tests comparing hierarchical models. A 5% significance level was applied.

Second, we tested the association between the seven neurocognitive functions and social functioning using an ordinal logistic regression model (proportional odds model). We categorized the ADOS-Total dependent variable, because the ADOS-Total did not meet the model assumption as continuous outcome, into the following categories: zero (no deviation), low (scores of 1 through 3), medium (scores of 4 through 6), and high (scores above clinical cutoff, i.e. ≥7). Age was entered as a covariate, group as a factor, and the seven neurocognitive functions were entered as independent variables or predictors. Interaction terms of each neurocognitive function with group were inspected. Only significant neurocognitive functions were retained in the final model. Finally, we repeated the first analysis while adjusting for the potential effects of depressive and anxiety symptoms by adding BDI-Y as covariates in one and BAI-Y in another reiteration of the repeated measures mixed effects model. We repeated the second analysis, by entering BDI-Y and BAI-Y separately as covariates in the ordinal logistic regression model. Prior to all analyses, four missing data points were replaced with the age-weighted group mean. Further, the raw scores of neurocognitive tests were transformed (by either square root, logarithmic or square root and reflect transformation) where appropriate. Extreme outliers were truncated to ±3SD from the sample mean, and all scores converted to z-scores (standardized), based on the age-weighted mean and SD of the control participants. We reversed z-scores for easier comparison across tests, in the tests where higher scores indicated poorer performance. For those neurocognitive functions measured with several single tests, we summed and re-standardized the included z-scores. Prior to combining test scores we inspected correlations between tests and confirmed that within each of the combined neurocognitive function z-scores, the included tests were significantly inter-correlated.

## Results

### Participants

Our sample comprised 43 participants with first-episode AN, 28 recovered participants, and 41 controls (Table 1). Individuals with first-episode AN were tested as close to admission date as possible; mean duration from first to last visit was 8.0 days (median 7.0 days, range 1–23 days, SD 5.8) and participants gained a mean of 2.1 kg from clinical assessment to first testing session (median 1.7 kg, SD 2.3). The two clinical groups did not differ regarding current comorbidity at time of study participation (Table 1a), and the three groups were comparable regarding intelligence and family background; however, mean age of participants with first-episode AN was lower (Table 1a). Three recovered participants currently used SSRI medication.

**Table 1:** Demographic and clinical characterization of participants with first-episode AN, participants recovered from AN, and controls

				Test statistics		Effect size^a^ (*p*)		
	FeAN (*N* = 43)	RecAN (*N* = 28)	Controls (*N* = 41)		*p*	FeAN vs. RecAN	FeAN vs. Con	RecAN vs. Con
1a At the time of study participation								
Age, mean (SD)	16.1 (1.5)	18.4 (1.6)	17.7 (2.2)	F(2, 109): 16.119	< .001	−1.48 (< .001)	−0.85 (< .001)	0.36 (ns)
Parents’ highest education, years (SD)	16.1 (2.3)	14.8 (2.8)	15.3 (2.4)	F(2, 109): 2.591	.08			
Living with both parents, N (%)	24 (56%)	17 (61%)	20 (49%)	Chi^2^(2) = .607	.74			
BMI (kg/m^2^) , mean (SD)	16.6 (1.2)	21.3 (1.8)	22.0 (2.6)	F(2,109): 92.608	< .001	−3.07 (< .001)	−2.67 (< .001)	−0.31 (ns)
BMI percentile corrected for age^b^, mean (SD)	7.5 (7.6)	47.3 (19.0)	56.9 (21.2)	F(2,109): 101.948	< .001	−2.75 (< .001)	−3.10 (< .001)	−0.48 (ns)
Comorbid depressive disorder^c,d^, N (%)	8 (19)	3 (11)		Fishers exact (2-sided)	.51			
Comorbid OCD, N (%)	3 (7)	0 (0)		Fishers exact (2-sided)	.27			
Comorbid anxiety other than OCD, N (%)	4(9)	5 (18)		Fishers exact (2-sided)	.30			
EDE global score, mean (SD)	2.8 (1.4)	0.6 (0.5)		t(54.33): 9.119	< .001	2.14		
RecAN duration of treatment (months)^e^, (SD)		23.9 (11.8)						
RIAS Intelligence Quotient, mean (SD)	107.7 (10.5)	102.8 (11.5)	107.3 (9.5)	F(2,109): 2.137	.12			
EDI^f^ Eating Disorder Risk Composite, mean (SD)	47.7 (10.1)	36.5 (6.4)	36.1 (7.0)	F(2,109): 24.861	< .001	1.32 (< .001)	1.33 (< .001)	0.06 (ns)
EDI Body Dissatisfaction Scale T-score, mean (SD)	49.0 (8.8)	37.9 (8.2)	36.9 (7.7)	F(2, 105): 25.254	< .001	1.31 (< .001)	1.46 (< .001)	0.13 (ns)
EDI Interoceptive Deficits Scale T-score, mean (SD)	49.4 (8.1)	35.3 (8.4)	36.0 (8.2)	F(2, 105): 34.794	< .001	1.71 (.001)	1.64 (< .001)	−0.08 (ns)
BYI Anxiety Index T-score, mean (SD)	57.1 (9.8)	51.8 (12.7)	47.8 (9.9)	F(2, 109): 8.092	.001	0.47 (ns)	0.94 (< .001)	0.35 (ns)
BYI Depression Index T-score, mean (SD)	60.9 (10.8)	49.8 (12.3)	48.5 (9.5)	F(2,109): 16.262	< .001	0.96 ( .001)	1.22 (< .001)	0.12 (ns)
1b At the time of treatment onset								
Age, mean (SD)	16.1 (1.5)	14.8 (1.6)		t(69) = 6.268	.001	0.84		
BMI percentile corrected for age, mean (SD)	3.84 (4.83)	4.83 (5.97)		t(69) = − .722	.47			
EDE global score at time of treatment^g^, mean (SD)	2.8 (1.5)	2.8 (1.3)		t(69) = − .051	.96			
Binge-purge subtype AN, N (%)	4 (9%)	4 (14%)		Fishers exact (2-sided)	.70			
Comorbid depressive disorder, N (%)	8 (19%)	5 (18%)		Fishers exact (2-sided)	.94			

*FeAN* first-epsiode AN participants, *RecAN* recovered AN participants, *Controls* control participants, *ns* non-significant (*p* < .05), *BMI* Body Mass Index, *EDI* Eating Disorder Inventory-3, *OCD* obsessive-compulsive disorder, *EDE* Eating Disorder examination, *RIAS* Reynolds Intellectual Assessment Scales

^a^Between-group effect sizes presented as Cohen’s d

^b^BMI percentiles corrected for age <0.02 are calculated as =0.02. Participants > 20 yr old were given percentile of 20 yr olds

^c^Depressive disorder includes mild depression, moderate depression and severe depression according to ICD-10.

^d^We used the term “comorbid” although anxiety and depression are only comorbid to AN in the case of the participants with first-episode AN

^e^We use months in treatment for the recovered as a proxy for duration of AN, knowing that this might not be entirely precise, because AN typically has a gradual onset

^f^EDI-3 answers are missing from two FeAN

^g^EDE data available from time of treatment for recovered participants, N = 13 (46%). T-scores are derived from American norms in the absence of Danish norms for adolescents

At time of treatment onset, the two clinical groups were comparable in terms of symptom characteristics (Table 1b), but the recovered participants were younger than first-episode AN participants at the time they entered treatment.

### Group differences in specific tests of neurocognition

**Fig. 1:**
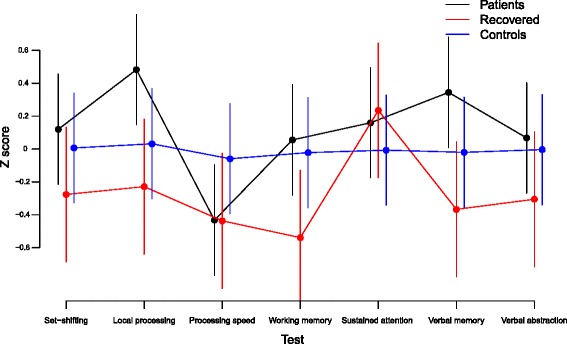
Profile plot, neurocognitive functions

The three groups displayed parallel profiles of neurocognitive functions (χ2 = 20.0, df = 12, p = .07) (Fig. 1).

Further, groups showed similar mean scores across neurocognitive functions and thus performance did not differ across groups (equal levels) (χ^2^ = 4.7, df = 2, p = .10). Scores on each neurocognitive function are presented for information in Table 2.

**Table 2:** Neurocognitive function z-scores

Neurocognitive function	FeAN (*N* = 43)		RecAN (*N* = 28)		Controls (*N* = 41)		Effect size^a^
	Mean (SD)	Range	Mean (SD)	Range	Mean (SD)	Range	R^2^
Set-shifting	0.31 (1.40)	(−2.55) -(3.48)	−0.37 (1.25)	(−2.48) -(1.62)	0 (1)	(−2.37) -(2.38)	0.05
Local processing	0.27 (1.06)	(−2.97) -(1.46)	−0.18 (0.88)	(−1.96) -(1.46)	0 (1)	(−2.18) -(1.46)	0.03
Working memory	0.02 (0.97)	(−2.28) -(2.24)	−0.52 (1.16)	(−2.67) -(1.20)	0 (1)	(−2.91) -(1.75)	0.05
Processing speed	−0.33 (1.10)	(−3.04) -(1.35)	−0.45 (0.96)	(−1.98) -(0.90)	0 (1)	(−3.65) -(1.66)	0.03
Sustained attention	0.02 (1.12)	(−2.71) -(2.10)	0.22 (0.98)	(−1.52) -(2.31)	0 (1)	(−2.48) -(2.34)	0.008
Verbal memory	0.21 (1.15)	(−2.05) -(3.10)	−0.47 (1.35)	(−2.05) -(3.02)	0 (1)	(−2.52) -(1.85)	0.05
Verbal abstraction	−0.06 (1.04)	(−2.58) -(2.16)	−0.16 (1.04)	(−2.58) -(1.65)	0 (1)	(−2.19) -(1.65)	0.004

Neurocognitive function: age-weighted z-scores, based on mean and SD of controls; *FeAN* first-episode AN participants, *RecAN* recovered AN participants, *Controls* control participants, *SD* standard deviation

^a^Age-weighted ANOVAs for each function separately, descriptive purposes only

### Effect of neurocognition on social functioning

Both of the clinical groups displayed lower levels of social functioning than controls (Independent-Samples Kruskal-Wallis Test: χ^2^(2) *=* 8.277, *p* = .02; first-episode AN: mean = 2.77, SD = 3.1, range = 0-10; recovered: mean = 2.79, SD = 3.2, range = 0-9; controls: mean = 1.05, SD = 1.5, range = 0-6) [5]. In an ordinal logistic regression predicting ADOS-Total categories, only the neurocognitive function of verbal memory was a significant predictor. Interactions between group and neurocognitive functions were not significant. Accordingly, a final model with group, age, and verbal memory as predictors yielded a Nagelkerkes pseudo-R^2^ of 21% (χ^2^(5) = 23.709, *p* < .0005). The odds of a higher ADOS-Total category were reduced to approximately half (Exp(B) = 0.584, 95% CI 0.423-0.808) with every increase in verbal memory (expressed as 1 point higher z-score)(Wald χ^2^(1) = 10.601, *p* = .001), and the final model predicted ADOS-Total significantly better than the simple model using only group and age as predictor (*p* < .001).

The odds of first-episode AN participants having a higher ADOS-Total classification were more than two times higher than controls in the final model, i.e., adjusted for verbal memory and age (Exp(B) = 2.490, 95% CI 1.013-6.117), Wald χ^2^(1) = 3.954, p = .05. The odds of recovered participants having a higher ADOS-Total classification compared with controls were three times higher (Exp(B) = 3.048, 95% CI 1.169-7.947), Wald χ^2^(1) = 5.195, *p* = .02.

The pattern of results was similar, when analysis was repeated excluding three participants using SSRI.

### Effect of depression and anxiety

The pattern of results for the analyses, both regarding group difference in neurocognition and effect of neurocognition on social functioning remained similar when adjusting for the possible confounding effects of depression and anxiety symptoms.

## Discussion

Young individuals with AN and those recovered from AN did not differ from controls in terms of their neurocognitive performance on the group level. However, we confirmed a positive association between social functioning and verbal memory across groups. Verbal memory contributed to the variance in social functioning above that accounted for by diagnostic group. The fact that diagnostic group remained a significant factor indicated that aspects of AN, other than verbal memory, also influenced the level of social functioning among individuals with AN.

Our findings extend the existing knowledge and document that the neurocognitive profile in young persons with AN cannot readily explain their difficulties with social functioning. The fact that deficits with social function were not limited to the acute state of illness, but persisted after recovery from adolescent AN, further suggests that starvation or other factors related to the acute state of AN do not fully explain the difficulties with social functioning in young persons with AN. Thus, future studies might benefit from focusing on temperamental and personality factors, as well as exploring the role of social anxiety or avoidance, and the interaction between these factors and adverse interpersonal processes during AN, as proposed by the interpersonal maintenance model of AN [4].

Reduced cognitive flexibility has been documented in adults with AN,[16] but not consistently in young persons with AN [22, 27, 53]. Reduced cognitive flexibility in individuals with AN has been associated with difficulties to adjust behavior in the context of a changing feedback [54, 55]. These observations informed the hypothesis of Zucker et al [19] concerning “*systemizing of social information*” as a compensatory strategy for individuals with AN in the face of difficulties in social interaction. However, clinical groups did not differ in flexibility from controls. Indeed, groups did not differ significantly on any measure of neurocognitive function, confirming recent reports of generally normal neurocognitive performance in children and adolescents with AN, [27, 32, 56, 57] in contrast to findings in adults with AN [16, 58, 59]. Small effect sizes of differences of neurocognitive function across groups in the current study further support a generally normal performance of the young clinical participants, rather than a type II error due to small sample size. Along the same lines, a meta-analysis of the Trail Making Test, one of several set-shifting tasks in our study, showed a negligible and non-significant effect size comparing young individuals with AN to controls [22]. However, it is not clear, whether the difference between adult and young samples might represent a “scar” related to longer duration of starvation in adults with AN, or whether the lower recovery rate in those with neurocognitive deficits may lead to a selection bias in adult samples. Finally, maturational effects may explain differences across age groups suggesting that abnormalities may not be evident or detectable until full maturation in adult age. This relevant topic requires further investigation in longitudinal studies.

Reduced verbal memory was associated with higher impairment of social functioning. It is noteworthy that the association between social functioning and verbal memory applied to all three groups, suggesting that verbal memory remains an important index for understanding the level of social functioning in general. In the absence of an interaction between group and verbal memory, this finding does not offer a disorder–specific explanation for impairments in social functioning. However, due to the smaller variance of ADOS-Total scores in the control group, the present association was mostly driven by the two clinical groups whose participants demonstrated a larger variance in ADOS-Total scores and in their level of verbal memory.

We assessed verbal memory with the “Memory for Stories” subtest, in which the stimuli were presented as a coherent narrative and challenged capacity of episodic rather than semantic memory. Episodic memory is the ability to learn, store, and retrieve information about unique experiences, and thus stores pieces of information together with the relations between them [60]. Performance in episodic memory benefits from associative encoding and retrieval [61]. Individuals of normal intelligence with ASD show normal memory for specific items, but are less likely to spontaneously recall relations between items [62]. Individuals with ASD thus tend to rely more on effortful executive control than on automatic associative processes in tasks of episodic memory [63]. In parallel, an impairment of associative encoding and retrieval [61] may contribute to the association between verbal memory and social function in our study population. Episodic memory is important for social function, especially for the context-sensitive regulation of interactions, because individuals need to keep track of the past behaviors of others they interact with [64].

Another line of evidence corroborates the fact that certain aspects of episodic memory may be affected in individuals with AN, namely the autobiographical memory recall, which is impaired in adults and adolescents with AN, along with a tendency to report overgeneralized memories [65–67]. Interestingly, both clinical groups in our study differed significantly from controls on the ADOS single items “*Offers Information*” and “*Communication of Own Affect*”, and the recovered participants further differed from controls on “*Reporting of Events*”[5] all of which evaluate the amount and quality of the participant’s propensity to share autobiographical information [6, 68].

We controlled for depressive and anxiety symptoms in the present study given their potential influence on social functioning [59, 69–71]. However, the association between social functioning and verbal memory was stable, even when adjusting for depressive and anxiety symptoms, discounting the hypothesis that these symptoms might underlie and confound the observed association.

### Strengths and limitations

The main strength of the current study is the comprehensive assessment of social functioning and neurocognition. Also, few studies among adolescents with AN have included recovered individuals as a contrast group to disentangle more stable traits from state factors related to the acute illness. To the best of our knowledge, potential associations between social function and neurocognition have not been studied in young individuals with first-episode AN, or in those fully recovered from AN. Some limitations, however, must be noted. First, the cross-sectional design of the present study cannot discern whether the observed impairments may reflect stable and potentially predisposing traits, or sequelae of AN. Second, our limited sample size did not allow subgroup analyses. Third, the test selection itself has several limitations. We cannot dismiss that several tests may measure more than one aspect of cognitive functioning, e.g. set-shifting and processing speed, and that the employed tests may not be sufficiently sensitive to detect subtle differences in these groups of participants. Further, the ADOS instrument was constructed to assess individuals with potential ASD. Future studies may benefit from developing more appropriate instruments to measure the subtle deficits of social functioning observed in the current population. Fourth, it would be preferable to assess the global integration aspect of central coherence skills in the same participants besides the local processing aspect. Fifth, while TOMAL Memory for Stories present coherent stimuli, the scores simply reflect the number of elements recalled. A scoring system rewarding cohesion of the recalled material might be more sensitive to associative encoding and recall ability. Sixth, even though we based our criteria for recovery on the existing literature*,* a higher threshold for normal weight and more objective data concerning the stability of normal body weight during the previous 12 months for recovered participants may have further strengthened our conclusions regarding this group. Last, the presence of three recovered participants using SSRI may be a confounder with effect on neurocognition.

## Conclusion

The clinical groups displayed generally normal neurocognitive functions. The neurocognitive function of verbal memory was associated with social functioning in all three groups. This association was robust when controlling for the effect of anxiety and depression. Verbal memory may provide a clue for understanding those who need additional interventions to improve social function. Individuals with lower verbal memory may benefit from strategies aimed at enhancing episodic memory. Psychoeducation for parents may prove beneficial if their child has a reduced verbal memory. For instance, psychoeducation on the specific role of verbal memory in social function may encourage parents to make connections between past and present verbal interactions more explicit. Psychoeducation may also help parents to plan careful exposures to help their child strengthen verbal memory in social contexts. Longitudinal studies may enhance our understanding of the development of social and neurocognitive functions and the interaction between the two functions among individuals with AN.

## Abbreviations

ADOS: Autism diagnostic observation schedule
AN: Anorexia nervosa
ASD: Autism spectrum disorders
BAI-Y: Beck anxiety inventory-youth (subscale of BYI)
BDI-Y: Beck depression inventory-youth (subscale of BYI)
BYI: Beck youth inventory
D-KEFS: The delis-kaplan executive function system
EDE: Eating disorders examination
GEFT: Group embedded figures test
ICD-10: International classification of diseases, 10th edition
K-SADS-PL: Schedule for affective disorders and schizophrenia for school-age children, present and lifetime version
MROAS: Morgan russell outcome assessment schedule
PASAT: Paced auditory serial addition test
RIAS: Reynolds intellectual assessment scale
SD: standard deviation
SSP: Spatial span
SWM: Spatial working memory
TOMAL: Tests of memory and learning
TOVA: Test of variables of attention
WAIS: The Wechsler adult intelligence scale
